# Analysis of crop disease and pest occurrences: Insights from Japan’s national surveys

**DOI:** 10.1371/journal.pone.0322579

**Published:** 2025-04-29

**Authors:** Jianqiang Sun, Sunao Ochi, Takehiko Yamanaka

**Affiliations:** 1 Research Center for Agricultural Information Technology, National Agriculture and Food Research Organization, Tsukuba, Ibaraki, Japan; 2 Institute for Plant Protection, National Agriculture and Food Research Organization, Tsukuba, Ibaraki, Japan; Graphic Era Institute of Technology: Graphic Era Deemed to be University, INDIA

## Abstract

Accurate forecasting of crop diseases and pests (CDPs) is crucial for ensuring food security. In Japan, nationwide CDP field surveys have been conducted for over half a century at an annual cost of approximately 300 million JPY, with the primary goals of predicting CDP outbreaks and estimating within-year damages. Despite the magnitude of these efforts, the collected data remain underutilized. Therefore, this study aimed to contribute to the advancement of this field by evaluating the potential of leveraging historical Japanese CDP survey data to forecast CDP occurrences. Through comprehensive analysis and statistical modeling, we found that a simple algorithm—averaging data from the past five years without incorporating seasonal trends or meteorological variables—outperformed more complex models, underscoring the value of historical CDP survey data. However, the prediction error remained substantial, with an RMSE of 6.2 ± 19.6. Notably, as 70.1% of the CDP survey data recorded values of five or less, an error of 6.2 indicates poor predictive accuracy in most cases. Given the challenges of precise forecasting, the high cost of nationwide surveys, and Japan’s lowest self-sufficiency rate, fundamental reforms are needed. Integrating modern technologies, including IoT/ICT and artificial intelligence, could enhance the sustainability of CDP survey, ultimately safeguarding food security.

## Introduction

The effective management of crop diseases and pests (CDPs) is critical for ensuring agricultural productivity and food security. Accurate forecasting of CDP impacts is essential for maintaining the quality and quantity of agricultural products and for the judicious use of agricultural chemicals, thereby protecting food security.

Since the 1940s, Japan has conducted nationwide field surveys of CDPs under guidance of the Ministry of Agriculture, Forestry and Fisheries (MAFF) of Japan. This initiative was introduced as part of a national policy to increase food production, with the goals of forecasting outbreak timings and quantifying the occurrence of CDPs [1]. Over the years, the survey system and methodology used have evolved to adapt to changing needs. Today, periodic fixed-point surveys are conducted in fields and greenhouses, primarily by plant pathologists and entomologists at prefectural pest and disease control institutes.

The survey results are required to be reported to the national government. Prior to 1996, the survey results were reported via mail and further achieved in books or digital documents in PDF format. These documents often featured inconsistent sentence and table structures that varied yearly and by prefecture, making modern digitization difficult. However, advances in communication technology during the 1990s enabled the establishment of an online reporting system and a centralized database, significantly improving the efficiency of data collection and management in the late 1990s [2].

This nationwide field survey costs approximately 300 million JPY annually, with historical peaks reaching as high as 1.2 billion JPY. Despite its long history, the survey now faces critical crossroads, including budget cuts, a reduction in workforce, and the increasing diversity of CDPs caused by climate change and evolving cultivation methods. Today, we have reached a milestone in evaluating whether the survey has achieved its initial objectives and in rethinking the survey methods used in the era of smart agriculture.

From a global perspective, Japan’s nationwide survey of CDPs stands out as unique. For more than half a century, no other country has allocated such a substantial government budget for conducting annual nationwide surveys. For example, in the United States, state governments and universities often lead efforts to collect meteorological data and predict CDP incidences at the state level [3]. Although online platforms, such as ipmPIPE (Integrated Pest Information Platform for Extension and Education) [4], have been developed for multiple states, they are managed by volunteers and collect limited crop development and pest-monitoring data. In Australia, the Grains Research and Development Corporation, as a part of the national organization, coordinates the research and development of agricultural technologies and conducts nationwide surveys of grain CDPs every decade [5]. Meanwhile, information on CDP surveys in European Union countries, as well as major agricultural exporters, such as India and Brazil, remains limited. These examples highlight the fact that comprehensive and long-term studies on crops and CDPs are unprecedented outside of Japan.

Data collected from the Japan nationwide field survey, hereafter referred to as MAFF CDP data, consist of the damage incidence (DI) and damage level caused by CDPs throughout Japan, and are categorized by crop, CDP, month, and prefecture. The DI is measured nearly every month during the growing periods for each crop and CDP combination, and is recorded as the percentage of damaged areas out of the total surveyed plant organs in fixed fields within each prefecture. For example, for crop diseases, depending on the growth stage of the crop, the percentage of diseased leaves, stems, flowers, and fruits are recorded. For pest damage, the percentage of damaged organs is recorded; if pests are parasitic to the plant at the egg stage, the percentage of parasitism is recorded. By contrast, the damage level is recorded on a scale from 1 (weak) to 5 (strong) based on a combination of the occurrence of CDPs over the past decade. The criteria for determining this level vary by prefecture, year, and CDP, making them unsuitable for quantitative analysis. Detailed survey methodologies are outlined in survey implementation standards published by the MAFF [6].

The objective of MAFF CDP data collection is to forecast the timing of CDP outbreaks and quantify their occurrence. However, aside from our previous study [7], which focused on four crops (cucumber, eggplant, strawberry, and tomato), limited efforts have been made to comprehensively analyze these records and scientifically quantify the occurrence of future damage, perhaps due to challenges related to data accessibility and analytical methodologies.

For instance, metadata describing the survey protocols for collecting the MAFF CDP data, as well as all non-numerical data, are written in Japanese. This linguistic barrier hinders the accessibility of the data for researchers worldwide, particularly in comparison to open data that are available in English-speaking countries. Furthermore, the data are collected at the prefectural level, with anonymized survey locations, including greenhouses, which complicates its integration with meteorological data for modeling. Moreover, variability in crop cultivation periods and durations introduces inconsistencies in the survey data, including gaps or missing values across different time intervals. These challenges pose significant barriers to adapting time-series algorithms for analysis.

Therefore, despite the implementation of various algorithms, including linear regression, Bayesian modeling approaches, and machine learning methods, such as random forests (RF), support vector machines, and neural networks, that have been successfully applied to practical pest management [8–11], most algorithms, except for RF, have proven insufficient when applied to the MAFF CDP data [7], highlighting the unique analytical challenges.

Here, we aimed to assess whether historical survey data on CDPs can be effectively used to forecast future CDP occurrences through comprehensive analysis and statistical modeling of the entire MAFF CDP survey data. We believe that our findings will provide unique insights that could inform the modernization of the nationwide field survey, enabling it to contribute to contemporary methodologies for quantifying future occurrences of CDP damage.

## Materials and methods

### MAFF CDP data

The incidence of crop and CDP combinations in the MAFF CDP data collected from March 1996 to December 2022 was used in this study. The original data record the percentage of specific organs (e.g., leaves, stems, flowers, and fruits) that were damaged by diseases, pests, and parasites out of all the organs surveyed. However, considering that no overlap occurred among the disease, pest damage, and parasitism rates, these three rates were referred to as the DI in this study so as to treat the MAFF CDP data collectively. For some important crops, multiple surveys had been conducted for the same crop and CDP combination within a month. In this case, the maximum value from multiple surveys was used as the monthly DI. This approach was adopted because predicting the maximum value of damage allows for a more accurate understanding of the severity of CDP spread, enabling prompt control measures. In summary, data containing the crop, CDP, year and month, prefecture, and DI information were screened out for further analysis.

### Meteorological data

Meteorological data from March 1996 to December 2022 were obtained from the Japan Meteorological Agency [12]. The website publishes historical meteorological records of the monthly average temperature, total precipitation, average humidity, total sunshine duration, and other statistics for each monitoring station. Typically, a single prefecture has multiple monitoring stations. In this study, multiple stations that uniformly covered a single, entire prefecture were selected. Then, averages of the monthly average temperature, total precipitation, average humidity, and total sunshine duration across the selected stations were calculated for each prefecture and used for the downstream analysis.

### Quantitative analysis of the damage incidence data

To analyze the composition and distribution of the DI, it was disaggregated across different levels: by crop, CDP, crop × CDP, and crop × CDP × prefecture. This allowed us to assess the distribution of sample sizes within each combination. In addition, to explore the relationships between the same CDP that affected different crops (e.g., the DI of cucumbers and strawberries damaged by the same powdery mildew disease), Pearson correlation coefficients (PCCs) were calculated for these combinations. Specifically, PCCs were calculated using a pair of DIs in which one was of a crop (e.g., cucumber) damaged by a CDP at a particular time point (year and month) in a prefecture and the other of another crop (e.g., other than cucumber) damaged by the same CDP at the same time point in the same prefecture. Data points were not included in the calculation if either DI value was missing. Furthermore, to identify potential monthly variations in the data, the DIs were grouped by month for each crop × CDP × prefecture combination and then used in an analysis of variance (ANOVA). The *p*-values obtained from multiple ANOVA testing were corrected (adjusted *p*-values) by controlling the false discovery rate using the Benjamini–Hochberg algorithm [13].

### Fitting and validation of the damage incidence

To forecast the DI for each crop × CDP × prefecture combination, three classic statistical algorithms—autoregressive integrated moving average (ARIMA), seasonal ARIMA (SARIMA), and SARIMA with exogenous factors (SARIMAX)—alongside two typical machine learning algorithms, Gaussian process regression (GPR) and RF (S1 Appendix), were employed. Additionally, three custom algorithms, past averages (PA), random values from a normal distribution (RAND), and random values from a log-normal distribution (LOGRAND), were applied. PA was designed as a simple model in which the average value of the past *n* years was used as the forecast value for the next year for each crop × CDP × prefecture combination. RAND and LOGRAND were designed to represent the null hypothesis. The predictions of RAND and LOGRAND were randomly sampled from normal and log-normal distributions, respectively, with the mean and standard deviation calculated from all time points for each crop × CDP × prefecture combination.

Fitting and validation were performed separately, considering the characteristics of the time-series data. For instance, to fit and validate the algorithm, the data for each crop × CDP × prefecture combination were divided into three parts: (i) from the earliest time point to December of year *y*; (ii) time points at *y* + 1 year; and (iii) time points after *y* + 1 year. The first subset was used as the training set for fitting, and the second used as the validation set to calculate the root mean squared error (RMSE). The year was incremented from the earliest to the latest years. The fitting and validation were repeated at each increment; thus, the RMSE was calculated at each *y*. Fitting and validation were omitted when the number of time points in the training subset was less than five. In addition, during the validation step, the RMSE was not calculated if the validation set had fewer than three time points because an RMSE calculated from a small sample is not reliable. After the repetition of all fitting and validation steps, the RMSEs calculated for all years were averaged. The average RMSE was recognized as the forecast performance of each crop × CDP × prefecture combination. The same procedures were performed for all crop × CDP × prefectures combinations.

The hyperparameters of ARIMA, SARIMA, and SARIMAX were computed within the default implementation of the R package, forecast [14], using the training set. To build the SARIMAX models, the monthly average temperature and total precipitation were used as the exogenous factors. GPR and RF were computed within the default implementation of the R packages, kernlab [15] and RandomForest [16], respectively, using the training set. For RF, the monthly average temperature and total precipitation were used as explanatory variables, and the DI as an objective variable. Additionally, the hyperparameter *n* of PA was computed via glid-search from 1–5.

## Results

### Diversity and sparsity of the MAFF CDP data

**Fig 1:**
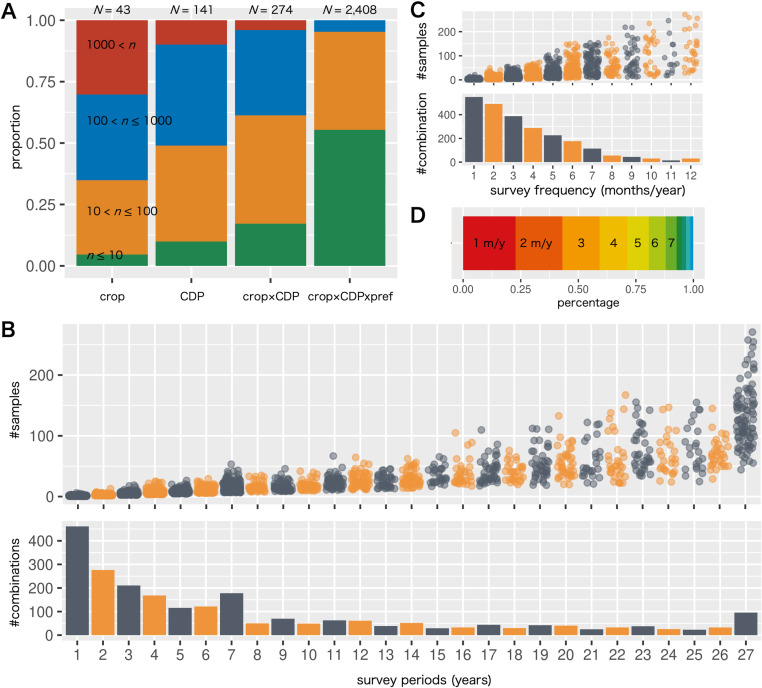
Overview of the diversity and sparsity of the MAFF CDP survey data. (A) Bar charts representing the proportion of the number of samples (n) at four levels: n ≤10 (green), 10< n ≤100 (orange), 100< n ≤1000 (blue), and n>1000 (red), for each category of crop, crop disease and pest (CDP), crop × CDP, and crop × CDP × prefecture. N indicates the number of types in each category. (B) Jittered points shown in the top panel indicate the relationship between survey period (years) and number of samples for each crop × CDP × prefecture combination. Bar charts in the bottom panel indicate the number of crop × CDP × prefecture combinations. (C) Jittered points shown in the top panel indicate the relationship between survey frequency (months/year) and the number of samples for each crop × CDP × prefecture combination. Bar charts in the bottom panel indicate the number of crop × CDP × prefecture combinations. (D) Bar chart representing the overall proportion of the survey frequency combinations.

The MAFF CDP data collected since 1996 cover a diverse range of 43 crops and 141 types of CDPs (Fig 1A, S1 Data). Crops included grains, vegetables, and fruit trees. Citrus had the largest number of samples, totaling 9,124 (i.e., 9,124 surveys or DI values), whereas more than half of the crops (69.8%) had fewer than 1,000 samples. The CDPs included 70 crop diseases and 71 pests, with the minority (9.9%) having more than 1,000 samples. When examining crop × CDP combinations, we found that only 11 (4.0%) combinations had more than 1,000 samples. Further, the crop × CDP × prefecture combinations had none with more than 1,000 samples, and a small percentage (4.7%) had more than 100 samples.

Next, we investigated the survey period and frequency for each crop × CDP × prefecture combination and found that the number of samples was influenced by the survey period length (Fig 1B, S1 Data) and frequency (Fig 1C, S1 Data). For instance, 19.1% of the combinations were surveyed for only one year, with most having three or fewer samples. In other words, the survey was conducted a maximum of three times in only one year. For survey periods of 5, 10, 15, 20, and 25 years, the sample counts ranged from 5–26, 10–42, 17–65, 20–133, and 25–155, respectively. The number of samples collected increased with an increase in the survey period. The lower limit was constrained by the inclusion of a single survey per year, whereas the upper limit theoretically allowed up to 12 surveys per year. However, in practice, more than half of the combinations had surveys conducted for three months or fewer each year, with approximately 80.6% for six months or fewer annually (Fig 1D), reflecting the fact that many crops are grown only for a few months each year.

The longest survey period in the MAFF CDP data (27 years) was conducted for 96 crop × CDP × prefecture combinations, representing 4.0% of all the combinations. These were predominantly combinations of important Japanese crops (e.g., tea, citrus, cucumber, and strawberry) and CDPs prevalent nationwide (e.g., spider mites, aphids, powdery mildew, and downy mildew). Survey frequency varied across prefectures. For example, cucumber downy mildew (*Pseudoperonospora cubensis*) has been surveyed 271 times over 27 years in Ehime, with the surveys conducted nearly every month, whereas in Kagawa and Kyoto, these were performed fewer than 90 times, approximately six times per year. Similarly, the citrus red mite (*Panonychus citri* McGregor) was surveyed in Miyazaki and Ehime ~250 times over 27 years, with surveys performed almost monthly, but only ~160 times in Chiba and Wakayama, approximately seven times a year. Despite the long survey period, the number of samples varied because different prefectures had different survey criteria and frequencies. This trend was consistent throughout the MAFF CDP data.

### Distribution of the damage incidences

**Fig 2:**
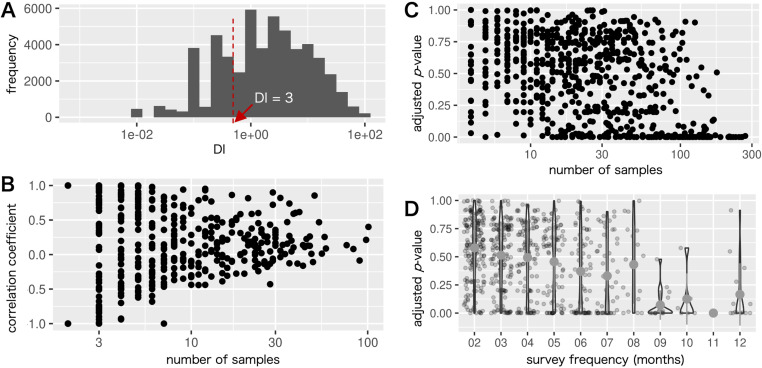
Distribution and characteristics of CDP incidences. (A) Distribution of the damage incidence (DI) in the MAFF crop disease and pest (CDP) survey data. (B) Relationship between the number of samples and Pearson correlation coefficient of pairs of the same CDP but with different crops in the same prefecture. (C) Relationship between the number of samples and adjusted *p*-value calculated using analysis of variance (ANOVA) for each crop × CDP × prefecture combination. (D) Relationship between the survey frequency and adjusted *p*-value calculated using ANOVA for each crop × CDP × prefecture combination.

The DI recorded in the MAFF CDP data followed a log-normal distribution, with an unnatural peak observed at specific round numbers (Fig 2A, S1 Fig in S1 Appendix). For instance, 3,386 (6.5% of all DI values), 2,747 (5.3%), 1,492 (2.9%), and 785 (1.5%) DI values were equal to 0.1%, 1%, 2%, and 3%, respectively. DI values at these round numbers, especially those that were ≤3%, were not continuous with their neighboring values, resulting in unnatural peaks in the distribution. DI values equal to or less than 1, 3, 5, and 10 accounted for 40.5%, 60.5%, 70.1%, and 81.8%, respectively.

### Correlations of the damage incidences between crops

To determine whether a CDP in a crop affects other crops in the same prefecture at the same time, PCCs of the DI values between different crops damaged by the same CDP (e.g., cucumber × powdery mildew and strawberry × powdery mildew) across all prefectures were calculated (Fig 2B, S2 Data). Owing to the shift in growing seasons, the PCCs for many combinations could not be calculated. Of the 486 PCCs, a large proportion (67.3%) was calculated from fewer than 10 paired points. Among the PCCs calculated from 10 or more samples, 25 (5.0%) and 12 (2.5%) pairs were satisfied with the criteria of 0.4≤ PCC <0.7 and PCC ≥0.7, respectively. The combinations that showed more than moderately positive correlations (i.e., PCC ≥0.4) included pests (e.g., *Scirtothrips dorsalis* and aphids) on different fruit trees (e.g., citrus, grapes, Japanese persimmon, and apples), diseases (e.g., mosaic diseases, powdery mildew, and downy mildew) among vegetables (e.g., cucumber, tomato, and green pepper), and diseases (e.g., panicle blast and rice stripe) and pests (e.g., *Chilo suppressalis* and *Cnaphalocrocis medinalis*) between early and ordinary seasonal rice plants. In contrast, in most pairs (92.5%), no or weak correlations (i.e., PCC <0.4) were confirmed between different crops affected by the same CDP, indicating that the DI of the CDP for one crop likely did not affect that of the other crops.

### Monthly trends of the damage incidences

An ANOVA was conducted to investigate monthly differences in the DI values for each crop × CDP × prefecture combination. Except for a large proportion of combinations unsuitable for ANOVA due to small sample sizes or data collected in a single month, 702 combinations were analyzed. Results showed that only 100 combinations satisfied the criterion of an adjusted *p*-value <0.01. The number of samples and survey frequency influenced the ANOVA results. Combinations with fewer samples or lower-frequency surveys tended to produce larger adjusted *p*-values. In contrast, significant monthly differences were more frequently observed in combinations with a larger number of samples (e.g., >150) (Fig 2C) or high-frequency surveys (e.g., more than eight monthly surveys per year) (Fig 2D).

### Fitting results of the damage incidences

Five classic statistical and machine learning algorithms (ARIMA, SARIMA, SARIMAX, GPR, and RF) and three custom algorithms (PA, RAND, and LOGRAND) were used to model the DI for each crop × CDP × prefecture combination. The fitting and validation processes considered the time-series data characteristics. For each combination, a validation RMSE was calculated. Among the 2,408 crop × CDP × prefecture combinations in the MAFF CDP data (Fig 1A), modeling was not conducted for 61.5% because of the insufficient number of samples that were available. For the remaining 927 combinations for which modeling was successfully conducted, the RMSE values computed from each modeling followed a log-normal distribution. Modeling results were categorized into three groups based on the length of the survey period: short-term surveys (STS) (≤5 years), medium-term surveys (MTS) (>5 and ≤10 years), and long-term surveys (LTS) (>10 years).

**Fig 3:**
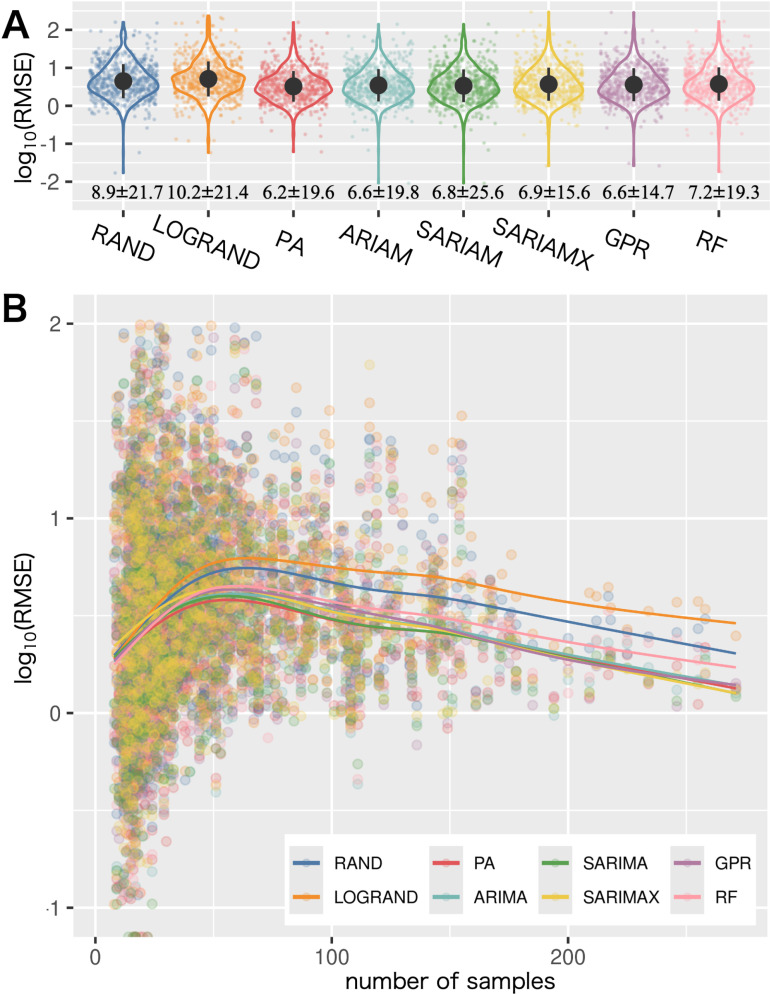
Modelling results of CDP incidences. (A) Violin charts with jittered points showing the root-mean-squared error (RMSE) distribution of the crop × crop disease and pest (CDP) × prefecture combinations for each algorithm. (B) Scatter chart representing the relationship between the number of samples and RMSE values. Regression curves fitted from both relationships are shown with solid lines for each algorithm. The colours of the points and lines indicate the type of algorithm.

The STS group included 33 combinations. Although the mean RMSEs of RAND and LOGRAND were lower than those of all the other models (S2 Fig in S1 Appendix), statistical significance was not confirmed (S1 Table in S1 Appendix). The MTS group (242 combinations) exhibited a similar trend (S2 Fig, S1 Table in S1 Appendix). In the LTS group (652 combinations), which used survey data obtained over more than 10 years, the mean RMSEs for all models were significantly lower than that of RAND (Fig 3A, S1 and S2 Tables in S1 Appendix).

In the LTS group, the PA model had an average RMSE of 6.2 ± 19.6, which was significantly different from that of the other algorithms (S1 and S2 Tables in S1 Appendix). The optimal hyperparameter *n* for PA was distributed as follows: *n* = 1 in 36.8% of cases; *n* = 2 in 17.0%; *n* = 3 in 11.9%; *n* = 4 in 10.4%; and *n* = 5 in 24.0%. The number of samples did not affect the hyperparameter *n* (S3B Fig in S1 Appendix). In addition, expanding the search range of *n* from one to all time points did not significantly alter the RMSE distribution (S4 Fig in S1 Appendix).

A regression analysis was conducted to examine the impact that the number of samples has on the RMSE values (Fig 3B). When the number of samples was below 100, the PA model tended to yield a lower RMSE than that of the other algorithms. In contrast, when the number of samples exceeded 100, most algorithms, except for RF, tended to yield similar RMSE values. The regression curves for RAND and the other algorithms, except for RF, were nearly parallel, particularly when the number of samples was greater than 100. This indicates that despite the increase in the number of samples, prediction errors between RAND (i.e., null hypothesis) and the other algorithms remained almost constant. In other words, the forecasting performance did not improve significantly as the sample size increased. RF, which relies on meteorological data for modeling, consistently showed slightly higher RMSE values than those of the other algorithms. Additionally, because longer survey periods and high survey frequencies tend to comprise a larger number of samples (Fig 1B and 1C), it might be thought that both factors theoretically affect the RMSE. However, no significant relationships were found between RMSE and the survey period (S5 Fig in S1 Appendix) or frequency (S6 Fig in S1 Appendix).

## Discussion

### Rationale of fitting for the crop and CDP combination

The MAFF CDP data encompass a wide range of crops and CDPs, yet the dataset is sparse (Fig. 1). Our analysis revealed that apart from a few CDPs that frequently occurred in crops with overlapping growing seasons, the presence of a particular CDP in one crop within a prefecture seldom influenced its presence in other crops (Fig 2B, S2 Data). Although a high correlation might be expected between pests capable of flying between crops, this was only confirmed for early and ordinary seasonal rice damaged by *Chilo suppressalis* and *Cnaphalocrocis medinalis*. These limited cases may be attributed to data sparsity or the crop specificity of certain pests (e.g., *Chilo suppressalis* only damages rice). Consequently, we concluded that developing a hierarchical model in which the hidden status of a CDP affects various crops within a prefecture is not particularly advantageous. Therefore, we modeled each crop × CDP combination independently in this study.

### Unimportance of meteorological data in fitting the damage incidences

The fitting results demonstrate that the simple PA model, which does not consider seasonal trends or meteorological data, outperformed the more complex models (e.g., SARIMA and SARIMAX). The RF model, which performed the best in our previous study wherein meteorological data were used [7], showed the worst performance when compared with that of the models used without meteorological data in this analysis. Similarly, SARIMAX, which incorporates additional meteorological data over SARIMA, did not significantly enhance the forecasting performance. These findings highlight the importance of historical field survey data on CDPs over meteorological data in predicting DI occurrences, even though meteorological conditions are known to influence CDP incidences [17,18].

Several factors may explain the diminished importance of meteorological data. CDP occurrence is influenced by the planting and growth stages of crops because CDPs do not occur in the absence of crops. For example, aphids and powdery mildew typically appear on cucumbers several months after planting. Therefore, the number of days elapsed since planting may be more critical than meteorological data are for DI modeling. The consistent planting schedules in each prefecture allowed the identification of monthly differences over several years, which may explain why models that capture historical monthly differences are better suited for DI forecasting.

Additionally, the alignment of meteorological data with MAFF CDP survey plots is inaccurate because exact field locations for the survey are kept confidential to protect the privacy of farmers. Although the averages of meteorological data collected from multiple monitoring stations that evenly covered a prefecture were used for modeling, they may not accurately reflect specific field conditions. In particular, crops cultivated in greenhouses, such as tomatoes, cucumbers, and strawberries, are more strongly affected by monthly differences rather than by meteorological data. Therefore, DI modeling using meteorological data alone is inadequate, and accumulated historical data are required.

Moreover, other reasons explaining the lower importance of the meteorological data may include the influence of soil conditions and CDP occurrences in previous years, which are not captured by meteorological data. Therefore, this suggests that PA, an algorithm that simply calculates monthly averages over the past several years, would be more effective than algorithms that use meteorological data are.

### Importance of the damage incidence over the past several years in fitting

Fitting the DI using PA without meteorological data, given that this exhibited the best performance, suggests that the number of years required for modeling depends on the specific crop × CDP combination. Optimization results of the hyperparameter *n* for the PA algorithm indicate that in some cases, data from the past year are sufficient (36.9% of cases), whereas in others, data from the past five years are required (23.8% of cases). As expanding the search range for *n* did not significantly improve the RMSE (S3 Fig in S1 Appendix), and no significant improvement was confirmed when the sample size was increased (Fig 3B), it is likely that having data from the past five years is adequate for constructing PA models. Additionally, models constructed with data spanning several decades (i.e., ARIMA, SARIMA, SARIMAX, and GPR) did not significantly outperform the PA models (Fig 3A, S1 and S2 Tables in S1 Appendix), suggesting that long-term data spanning several decades may not be necessary for forecasting the DI.

### Fitting evaluation

The fitting results are evident of the fact that prediction errors of the models created by the statistical and machine learning algorithms are better than those of the random algorithms (i.e., RAND and LOGRAND). However, the absolute prediction error was substantial, with the PA model averaging an RMSE of 6.2 ± 19.6. Considering that 70.1% of the entire MAFF CDP data had a DI of 5 or less, an error of 6.2 indicates an inaccurate prediction in most instances. Despite the overall superiority of PA models, the MAFF CDP data may be challenging to use for precise forecasting but could still provide some value in understanding trends in normal-year increases and decreases.

Additionally, due to the uniqueness of MAFF CDP data, no prior studies have been conducted that are directly comparable with our research. To the best of our knowledge, many studies on CDP outbreak modeling focused on specific crop × CDP combinations and leveraged extensive datasets [8–11]. Consequently, comparing the results of this study with those of prior research and evaluating the methodology using the same criteria are extremely difficult.

### Prospects for the nationwide field survey of crop diseases and pests

Given the challenges of forecasting CDP incidence using current MAFF CDP data and the difficulties in conducting and using nationwide surveys, the following question arises: is it necessary to continue nationwide CDP surveys? We conclude that even if MAFF CDP data cannot be used for precise future predictions, they remain valuable for understanding the general trends in CDP incidences. Specifically, these data reflect the current status of CDP incidences at the time of the survey and can help in sharing information to prevent major outbreaks and minimize the impact of CDPs on surrounding areas. Therefore, the continuation of CDP surveys is crucial. However, fundamental reforms are required to adopt modern technologies, including the Internet of Things, information and communication technologies, and artificial intelligence.

It is important to develop and operate a CDP-monitoring application system that can be used not only by the government and researchers but also by companies and farmers. The system could incorporate survey results from experts in plant pathology and entomology in each prefecture, including drone images, photos of crops, images from sticky pest traps, and monitoring videos from pest traps. Additionally, the system could include images and text information uploaded by farmers. The backend of the system could employ the latest technologies, including artificial intelligence, to analyze these data quickly and comprehensively, involving experts as needed, to provide current status updates and short-term forecasts of CDP incidence. This information would be promptly communicated to plant protection departments and farmers to aid in implementing control measures. Sustainable system operation requires stable support from government agencies and easy data sharing with research institutions.

In Japan, over 60% of food is imported [19], and its food self-sufficiency rate is the lowest among the major developed countries. To safeguard food security, it may thus be necessary to initiate another agricultural revolution through concerted efforts between public and private sectors.

## Supporting information

S1 Data An Excel file consisting of a detailed composition of the combinations and the number of samples in each category: crop, crop disease and pest (CDP), crop × CDP, and crop × CDP × prefecture. Additionally, information on survey periods, frequencies (months/year), and the start and end years of the surveys for each crop × CDP × prefecture combination were recorded.
(XLSX)

S2 Data An Excel file consisting of Pearson correlation coefficients (CPPs) of the disease incidence (DI) values between different crops damaged by the same crop disease and pest (CDP). The positive (CPP ≥0.7) and moderate (0.7> CPP ≥0.4) correlations are highlighted in red and yellow, respectively.
(XLSX)

S1 Appendix A PDF file consisting of supplementary figures and tables. (PDF)
