# Supplementary material for: Analysis of crop disease and pest occurrences: Insights from Japan’s national surveys
Source: PLoS One. 2025 Apr 29;20(4):e0322579. doi: 10.1371/journal.pone.0322579 (PMC12040168; doi:10.1371/journal.pone.0322579)
Supplement: S1 Appendix — (PDF) [file pone.0322579.s003.pdf]

SUPPLEMENTAL MATERIAL

Contents:

S1. Characteristics of fitting algorithm

Figure S1. Estimation of the distribution of damage incidences

Figure S2. Distribution of RMSE

Figure S3. Characteristics of hyperparameter *n* in PA

Figure S4. Distribution of RMSE of PA and PA with the extended search range

Figure S5. Relation between the survey periods and RMSE

Figure S6. Relation between the survey frequency and RMSE

Table S1. Statistical test (paired *t*-test) for RMSE distributions

Table S2. Statistical test (paired *t*-test) for RMSE distributions

S1. Characteristics of the fitting algorithm

Autoregressive integrated moving average (ARIMA) can model a wide range of time-series data patterns and makes forecasts based on past data by combining three components: autoregressive (AR), integrated (I), and moving average (MA). The AR component models the relationship between an observation and the number of previous observations, I explains the constrained data stationarity by removing trends in the data, and MA smooths out the data by averaging the residual past errors. ARIMA fitting and forecasting were performed using the following codes:

```
# fitting

m <- auto.arima(DI, ic = 'aic', stepwise = TRUE, approximation = FALSE,
        start.p = 1, start.q = 1, max.p = 5, max.q = 5, max.order = 10)

# forecasting

forecast::forecast(m, h = h)
```

Seasonal ARIMA (SARIMA) adds a seasonal component to ARIMA and is useful for capturing seasonal trends. The fitting and forecasting with SARIMA were performed using the following codes:

```
# fitting

DI <- ts(DI, frequency = t)

m <- auto.arima(DI, ic = 'aic', stepwise = TRUE, approximation = FALSE,
        seasonal = TRUE,
        start.p = 1, start.q = 1, max.p = 5, max.q = 5, max.order = 10)

# forecasting

forecast::forecast(m, h = h)
```

SARIMA with exogenous factors (SARIMAX) is an advanced version of SARIMA that can handle external influences (e.g., temperature and precipitation), making it a powerful tool for more complex time-series forecasting. The fitting and forecasting with SARIMAX were performed using the following codes:

```
# fitting

DI <- ts(DI, frequency = t)

m <- auto.arima(DI, ic = 'aic', stepwise = TRUE, approximation = FALSE,
        seasonal = TRUE,
        start.p = 1, start.q = 1, max.p = 5, max.q = 5, max.order = 10,
        xreg = meteorological_data)

# forecasting

forecast::forecast(m, h = h, xreg = meteorological_data)
```

Gaussian process regression (GPR) is a machine-learning algorithm used to predict values based on known data. It can be used to estimate smooth curves that best fit known data points and to use this curve for prediction. Fitting and forecasting with GPR were performed with the following codes, using the “kernlab” package.

```
# fitting
```

```
m <- kernlab::gausspr(seq_along(DI), DI, type = 'regression',
         scaled = TRUE, kernel = 'rbfdot', kpar = 'automatic',
         variance.model = TRUE)

# forecasting

kernlab::predict (m, h, type = 'response')
```

Random forest (RF) works by combining the results of many decision trees to make a final prediction. The decision tree is considered a flowchart that helps the machine make a decision based on a series of questions (i.e., explanatory variables, including temperature and precipitation). Fitting and forecasting with RF were performed with the following codes, using the “RandomForest” package.

```
# fitting

m <- randomForest::randomForest(x = meteorological_data, y = DI,
        type = 'regression')

# forecasting

randomForest::predict (m, meteorological_data)
```

The PA was designed as a simple model in which the average value of the past *n* years was used as the forecast value for the next year for each combination of crops and CDP in each prefecture.

The RAND model was designed to test this hypothesis. The prediction of RAND was randomly sampled from a normal distribution, with the mean and standard deviation calculated from all time points for each combination of crop and CDP in each prefecture.

Figure S1. Estimation of the distribution of damage incidences

The histogram of damage incidence (DI) values represented a distribution similar to a log-normal distribution with several unnatural peaks (Figure 1B). To accurately examine the distribution of the DI, we assumed that the data followed a normal, log-normal, and gamma distribution and performed fitting using the maximum likelihood method. Fitting was performed with "fitdistrplus" package. The estimated theoretical densities, empirical and theoretical cumulative distribution functions (CDF), Q-Q plots, and P-P plots are shown in the following figure. The shape of the theoretical density indicated that the DI values fit the log-normal and gamma distributions. In particular, for small DI values, the gamma distribution provided the best fit. In addition, the theoretical CDFs and P-P plots show that the log-normal and gamma distributions are candidates for fitting the DI. Furthermore, the Akaike information criteria (AICs) for fitting the normal, lognormal, and gamma distributions were 405814.4, 266901.2, and 272904.3, respectively. Overall, the results indicate that the DI fits a log-normal distribution.

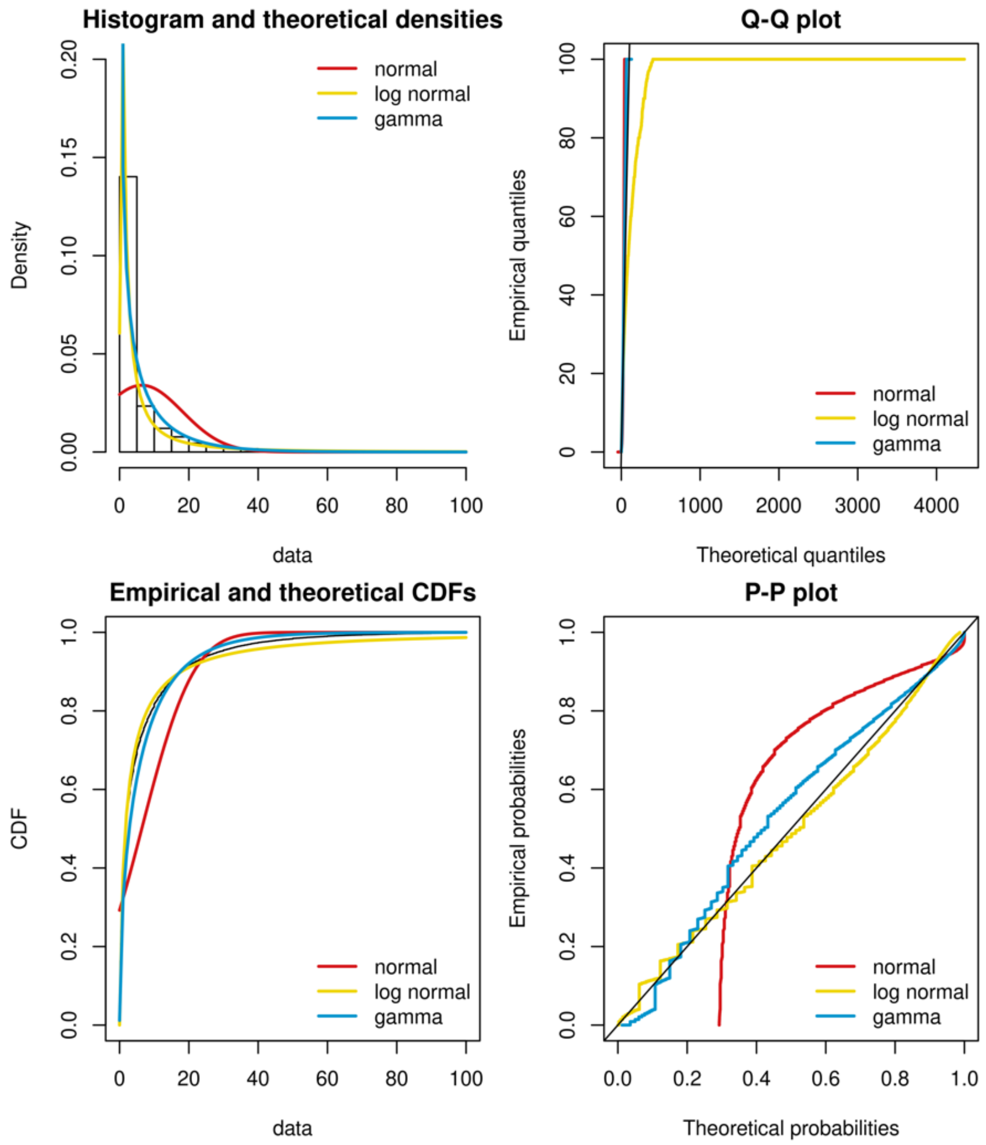

Figure S2. Distribution of RMSE

The distribution of RMSEs is represented by violine charts and box charts with jittered points grouped by data composed of short-term surveys (STS), medium-term surveys (MTS), and long-term surveys (LTS). The mean ± standard deviation of the RMSE distribution is shown in each chart.

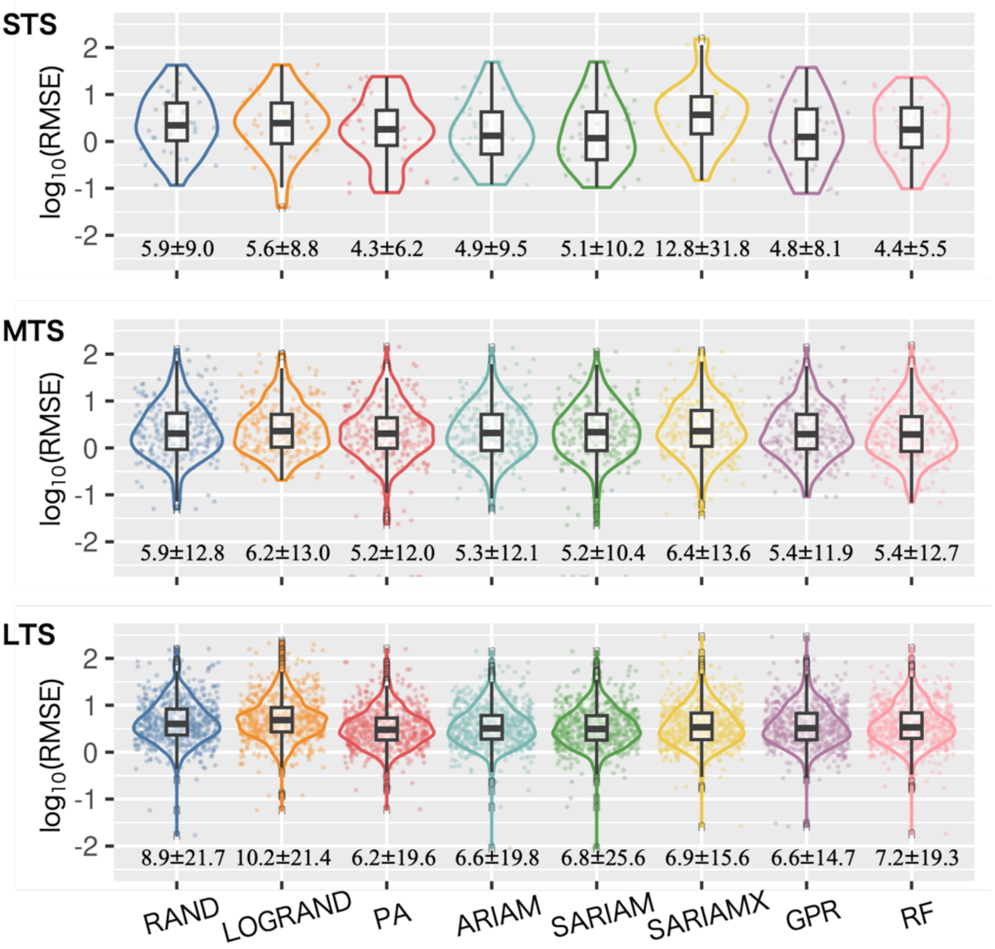

Figure S3. Characteristics of hyperparameter *n* in PA

The hyperparameter *n* was determined with a Glid search within the range of one–five. The optimal hyperparameter n for PA was found to be distributed as follows: n = 1 in 36.8% of cases, n = 2 in 17.0%, n = 3 in 11.9%, n = 4 in 10.4%, and n = 5 in 24.0%. Subplot **A** represents the relationship between the number of samples and the hyperparameter *n* using jittered points, indicating that no correlation was observed between the two variables. This suggests that the optimal *n* may depend on the specific crop–CDP combination. Additionally, subplot **B** represents the relationship between the hyperparameter *n* and the RMSE, suggesting that *n* does not affect the fitting performance.

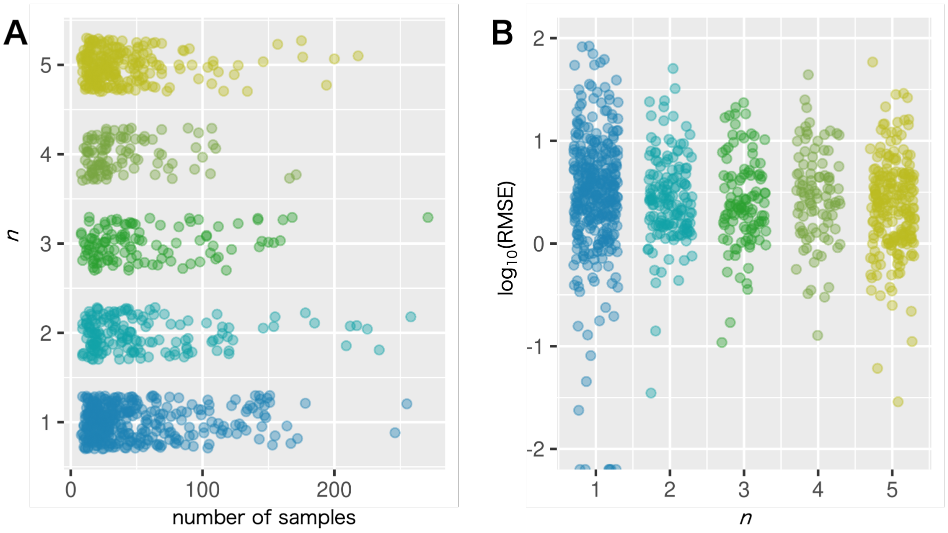

Figure S4. Distribution of RMSE of PA and PA with the extended search range

The hyperparameter *n* was determined with a Glid search within the range of one–five. To examine the influence of samples from the past five years, we extended the grid search range from one to ten and including *N* (all past time points) (PAX; PA with an extended search). Subplot **A** shows the distributions of the RMSEs for the PA and PAX models using box charts with jitter points. Subplot **B** represents the relationship between the number of samples and RMSE for the PA and PAX models. The results demonstrate that both PA and PAX produce similar RMSE distributions. The paired *t*-test indicated no significant difference in the mean RMSE distributions between the PA and PAX groups (p = 0.5542).

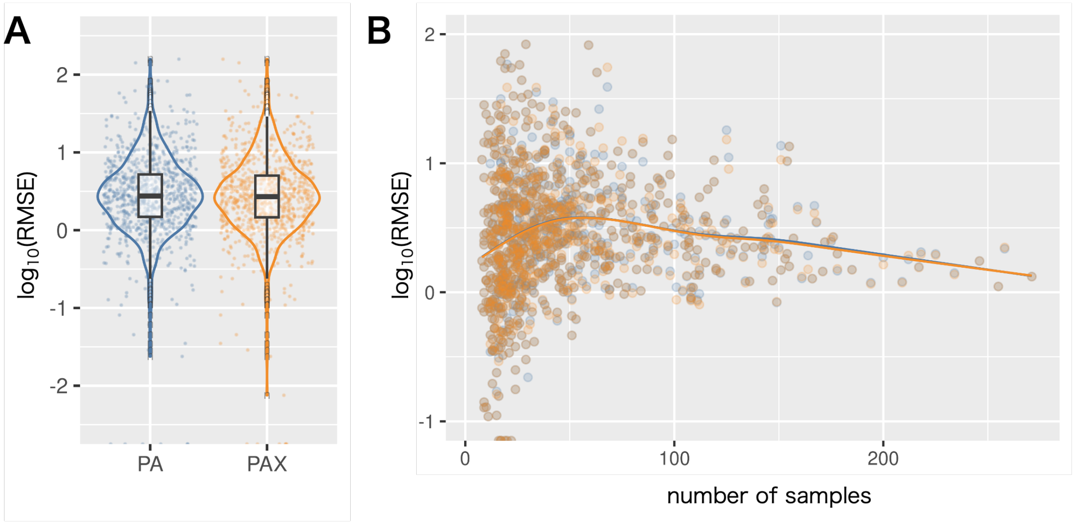

Figure S5. Relation between the survey periods and RMSE

Each subplot represents the relationship between the survey periods (years) and RMSE.

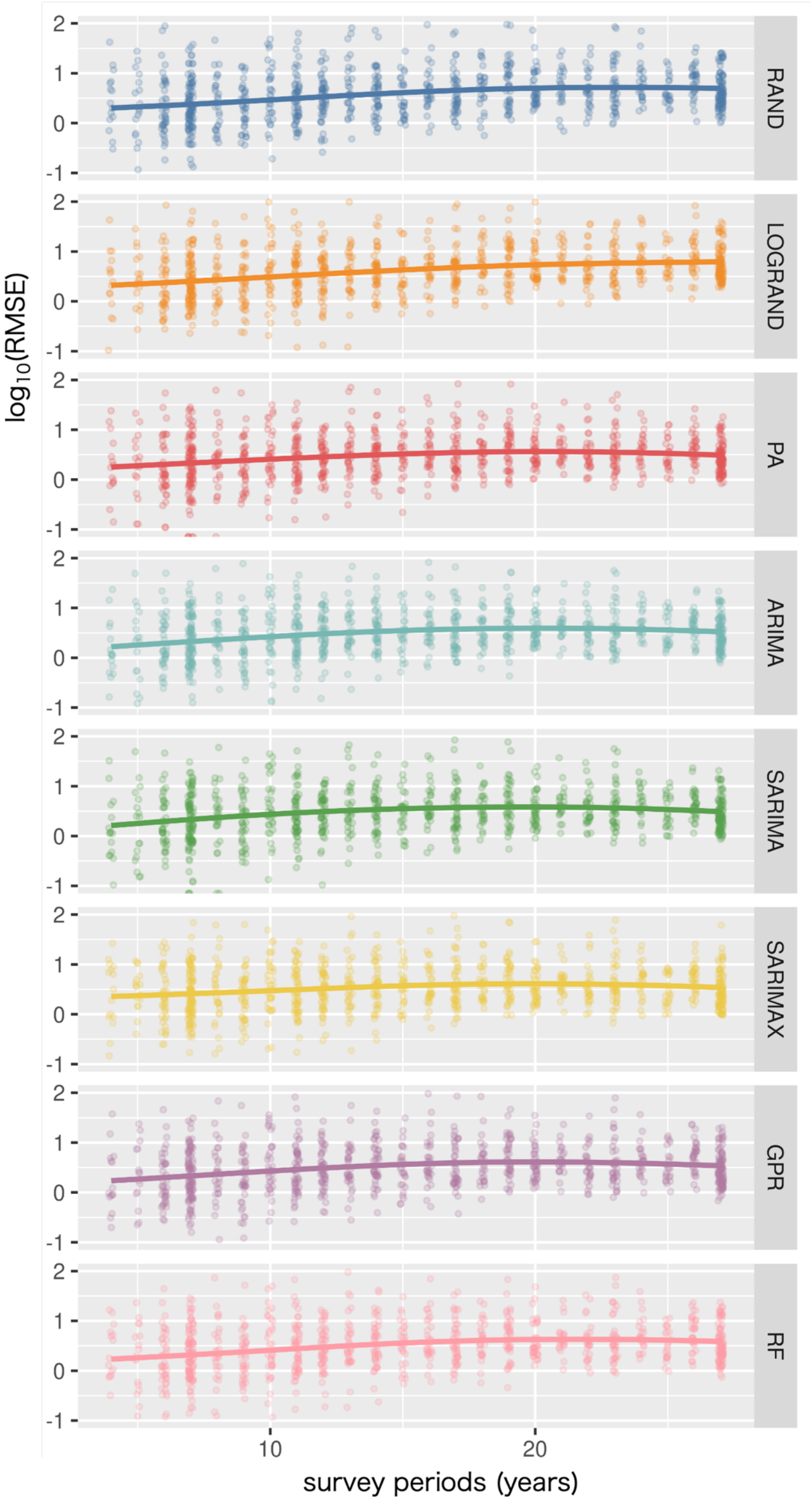

Figure S6. Relation between the survey frequency and RMSE

Each subplot represents the relationship between the survey frequency (i.e., number of surveys per a year) and RMSE.

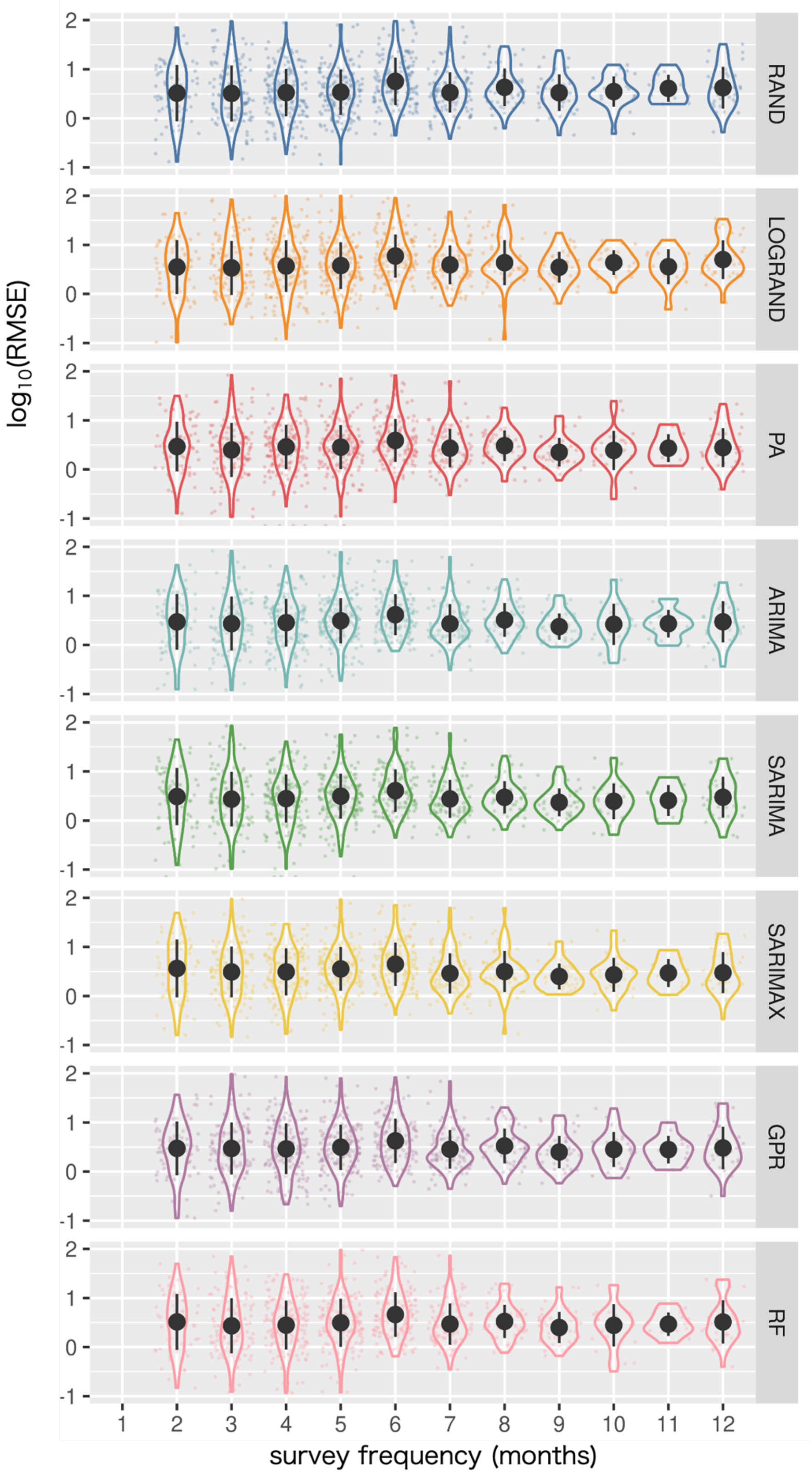

Table S1. Statistical test (paired *t*-test) for RMSE distributions

Paired *t*-tests were performed for all model combinations in each group. The *p*-values for the statistical results are listed in table. The *p*-value less than 0.01 is represented with red color.

Group	Model	RAND	PA
STS	**LOGRAND**	0.34231	
	**PA**	0.28984	
	**ARIMA**	0.30946	0.83716
	**SARIMA**	0.21166	0.39989
	**SARIMAX**	0.03566	0.00019
	**GPR**	0.20575	0.49279
	**RF**	0.67694	0.17106
MTS	**LOGRAND**	0.00003	
	**PA**	0.00274	
	**ARIMA**	0.06115	0.02730
	**SARIMA**	0.03468	0.13975
	**SARIMAX**	0.08119	0.00001
	**GPR**	0.51582	0.00279
	**RF**	0.12754	0.00527
LTS	**LOGRAND**	3.7.E-15	
	**PA**	1.6.E-39	
	**ARIMA**	8.7.E-28	1.2.E-04
	**SARIMA**	9.8.E-28	2.7.E-02
	**SARIMAX**	1.8.E-14	7.1.E-11
	**GPR**	9.3.E-24	1.5.E-07
	**RF**	7.0.E-20	8.1.E-18

Table S2. Statistical test (paired *t*-test) for RMSE distributions

Paired *t*-tests were performed for all model combinations for the MTS and LTS groups, which consisted of more than 200 combinations. To reduce the impact of sample size on the test results, we randomly sampled 200 combinations for testing. The *p*-values for the statistical results are listed in table. The *p*-value less than 0.01 is represented with red color.

Group	Model	RAND	PA
MTS	**LOGRAND**	0.00043	
	**PA**	0.01243	
	**ARIMA**	0.13712	0.00559
	**SARIMA**	0.09540	0.04369
	**SARIMAX**	0.04340	0.00005
	**GPR**	0.80897	0.01221
	**RF**	0.26267	0.00789
LTS	**LOGRAND**	3.5.E-04	
	**PA**	5.2.E-12	
	**ARIMA**	8.6.E-11	2.9.E-03
	**SARIMA**	4.4.E-09	2.8.E-01
	**SARIMAX**	4.7.E-04	1.9.E-07
	**GPR**	1.4.E-08	2.2.E-02
	**RF**	2.4.E-10	2.3.E-03
